# Genome-Wide Transcriptome Analysis Reveals Conserved and Distinct Molecular Mechanisms of Al Resistance in Buckwheat (*Fagopyrum esculentum* Moench) Leaves

**DOI:** 10.3390/ijms18091859

**Published:** 2017-08-27

**Authors:** Wei Wei Chen, Jia Meng Xu, Jian Feng Jin, He Qiang Lou, Wei Fan, Jian Li Yang

**Affiliations:** 1 Research Centre for Plant RNA Signaling , College of Life and Environmental Sciences, Hangzhou Normal University, Hangzhou 310036, China; chenweiwei@hznu.edu.cn; 2 Global Institute for Food Security, University of Saskatchewan, Saskatoon, SK S7N 4J8, Canada; 3 State Key Laboratory of Plant Physiology and Biochemistry, College of Life Sciences, Zhejiang University, Hangzhou 310058, China; 11507030@zju.edu.cn (J.M.X.); lxm0404@zju.edu.cn (J.F.J.); 0015631@zju.edu.cn (H.Q.L.); 4 College of Resource and Environment, Yunnan Agricultural University, Kunming 650201, China

**Keywords:** aluminum toxicity, buckwheat, cell wall, chromatin modification, transcription regulation, transporter

## Abstract

Being an Al-accumulating crop, buckwheat detoxifies and tolerates Al not only in roots but also in leaves. While much progress has recently been made toward Al toxicity and resistance mechanisms in roots, little is known about the molecular basis responsible for detoxification and tolerance processes in leaves. Here, we carried out transcriptome analysis of buckwheat leaves in response to Al stress (20 µM, 24 h). We obtained 33,931 unigenes with 26,300 unigenes annotated in the NCBI database, and identified 1063 upregulated and 944 downregulated genes under Al stress. Functional category analysis revealed that genes related to protein translation, processing, degradation and metabolism comprised the biological processes most affected by Al, suggesting that buckwheat leaves maintain flexibility under Al stress by rapidly reprogramming their physiology and metabolism. Analysis of genes related to transcription regulation revealed that a large proportion of chromatin-regulation genes are specifically downregulated by Al stress, whereas transcription factor genes are overwhelmingly upregulated. Furthermore, we identified 78 upregulated and 22 downregulated genes that encode transporters. Intriguingly, only a few genes were overlapped with root Al-regulated transporter genes, which include homologs of *AtMATE*, *ALS1*, *STAR1*, *ALS3* and a divalent ion symporter. In addition, we identified a subset of genes involved in development, in which genes associated with flowering regulation were important. Based on these data, it is proposed that buckwheat leaves develop conserved and distinct mechanisms to cope with Al toxicity.

## 1. Introduction

Acidic soils comprise approximately 50% of potentially arable lands worldwide, and only 4.5% of the total acidic soil area is exploited for agriculture [1]. Among the various factors limiting growth in acidic soils, the high Al concentrations are considered the major constraint to plant productivity [2]. Nonetheless, there is a great variation among different plant species, or cultivars within the same species, in resistance to Al toxicity [3,4].

Al resistance refers to the ability of the plant to maintain productivity at elevated Al concentrations, relying on either Al avoidance or Al tolerance [5]. In general, most crop plants resist Al toxicity by means of Al avoidance, whereby Al is excluded from the growing root tip through exudation of Al-chelating ligands such as organic acid anions. Thus, the Al concentration in aboveground parts is very low (<100 µg·(g·dry·wt)^−1^) [6,7,8]. Species that accumulate Al, by contrast, can accumulate more than 1000 mg·kg^−1^ A1 in the leaves [7]. For example, Matsumoto et al. reported that tea (*Camellia sinensis*) plants grown in the field can accumulate 30,000 mg·kg^−1^ A1 in old leaves, although only 600 mg·kg^−1^ A1 in young leaves [9]. After 6 weeks cultured in a nutrient solution containing 0.5 mM Al, *Melastoma malabathricum* accumulated 10,000 mg·kg^−1^ Al and 7000 mg·kg^−1^ Al in the old and young leaves, respectively [10]. Hydrangea (*Hydrangea macrophylla*) plants can accumulate 5000 mg·kg^−1^ Al in the leaves [11]. However, the molecular mechanisms by which these plant species accumulate and tolerate high concentrations of Al in leaves remain poorly understood.

Buckwheat (*Fagopyrum esculentum* Moench) grows well in acidic soils by means of different strategies to deal with Al toxicity. Buckwheat secretes oxalate rapidly from the root tip, in response to Al toxicity, to chelate rhizosphere Al and prevent Al entry into the root tip cells [12,13,14]. Once taken up, Al is bound as an Al-oxalate complex that is either compartmentalized into vacuoles or is transported radially into the xylem. Thus, buckwheat has evolved both avoidance and tolerance mechanisms to cope with Al toxicity in roots. In addition, buckwheat is characterized as an Al accumulator [15]. Buckwheat leaves accumulate more than 400 mg·kg^−1^ Al after 5 days cultured in nutrient solution containing Al [15], and as much as 15,000 mg·kg^−1^ Al when grown in an acidic soil [16]. Thus, in addition to Al resistance in the root, buckwheat is also able to accumulate Al within leaves, thereby resisting Al in the leaves. These features rank buckwheat as an excellent model for unravelling Al resistance mechanisms in plants, which will be important for the genetic improvement of Al resistance in other crops through biotechnological approaches.

Recently, Yokosho et al. carried out transcriptome analysis of buckwheat leaves in response to short-term moderate Al stress, and singled out possible transporter genes involved in Al resistance in buckwheat leaves [17]. Among 25 candidates, only *FeSTAR1* (sensitive to Al rhizotoxicity 1) might be involved in Al resistance by interacting with *ALS3* (aluminum sensitive 3) to protect the cell wall from damaging Al interactions [18]. Therefore, it remains largely unknown how buckwheat leaves accumulate and tolerate Al. 

In the present study, we carried out genome-wide transcriptome analysis of Al-regulated genes in buckwheat leaves. We identified a total of 33,931 unigenes from buckwheat leaves. Functional classification of Al-responsive genes revealed critical events involved in the adaptation to Al stress in buckwheat leaves. By comparing genes encoding transcription factors (TFs) and transporters between roots and leaves, we not only reveal some conserved mechanisms of Al resistance, but also suggest candidate genes which play distinct roles in these mechanisms.

## 2. Results

### 2.1. Buckwheat Leaves Rapidly Accumulate Al

In order to carry out RNA sequencing (RNA-seq) of buckwheat leaves in response to Al stress, we first examined the ability of buckwheat leaves to accumulate Al. To reach this purpose, we compared Al concentrations in roots and leaves between rice bean and buckwheat. Rice bean is an Al excluder that does not accumulate Al in aboveground parts [19], but buckwheat is an Al accumulator [15]. There was a little amount of Al in the roots and shoots in the control treatment, but no differences were observed between rice bean and buckwheat (Figure S1). After treatment with 20 µM Al for 24 h, both rice bean and buckwheat accumulated a substantial amount of Al in their roots (Figure S1A). It seems that buckwheat roots accumulated slightly less (but not significantly less) Al than did rice bean roots (Figure S1). However, the Al concentration in buckwheat leaves was about six times higher than that of rice bean (Figure S1B), confirming that buckwheat can efficiently translocate Al from roots to leaves. We have previously demonstrated that an Al concentration of 20 µM results in the moderate inhibition of root elongation [20]. Thus, we employed an Al concentration of 20 µM Al and a treatment time of 24 h for the construction of RNA-seq libraries.

### 2.2. De Novo Assembly of the Transcripts and Annotation

After quality control, we obtained approximately 89.5 and 59.1 million clean reads with a mean length of 80 bp from the leaves of the control (−Al) library and Al-treated (+Al) library, respectively (Table 1). The complementary DNA sequence was de novo assembled based on Trinity software, because the buckwheat genome remains unsequenced [21]. We assembled a total of 50,782 transcripts with a mean length of 716 bp, and 33,931 unigenes with a mean length of 677 bp (Table 1). BLASTx was adopted to annotate unigenes in the NCBI non-redundant protein (Nr) database, with a cut-off *E*-value of 10^−5^. The result was a total of 26,300 unigenes (77.96%) with high sequence identity to known genes (Table S1).

### 2.3. Global Effect of Al Stress on Gene Expression

The changes in the expression of the unigenes was calculated by the reads per kilobase of exon per million mapped reads (RPKM) method [22]. To understand the effects of Al stress on global gene expression in buckwheat leaves, those genes with log_2_ FC (fold change) ≥ 1 and with absolute values of RPKM ≥ 10 were considered as differentially expressed genes (DEGs). On this basis, a total of 2007 unigenes were considered as DEGs under Al stress, with 1063 and 944 unigenes being up- and downregulated, respectively (Table S2). 

**Figure 1:**
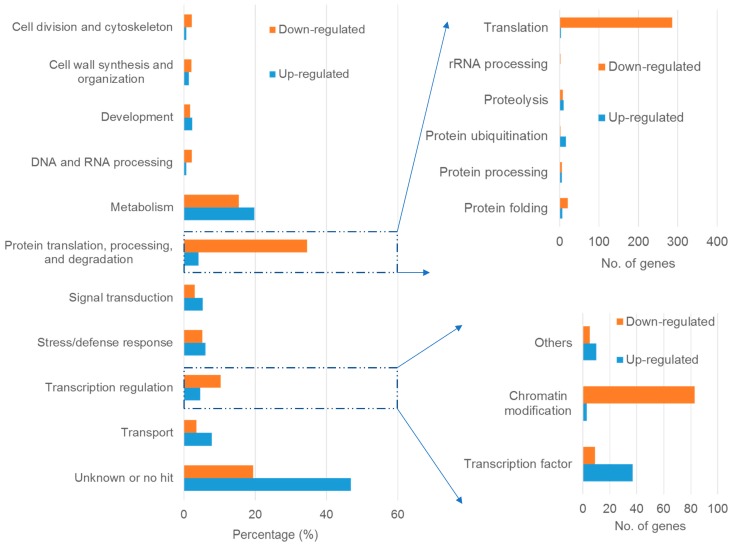
Functional categories of up- and downregulated DEGs. The categorization was performed according to the gene ontology (GO) biological process. The percentage or number of genes in each category is shown.

To obtain an overview of these DEGs, BLAST analysis was performed against The Arabidopsis Information Resource (TAIR) database (Available online: http://www.arabidopsis.org/Blast/index.jsp), such that each gene homolog was classified into a specific functional category based on the TAIR gene ontology (GO) biological process (Figure 1). Of the upregulated DEGs, 46.8% were annotated as unknown function or had no hit, and of the downregulated DEGs, 19.4% were annotated as unknown function or had no hit. In the upregulated DEGs, ‘metabolic process’ was the most represented category, which is consistent with our RNA-seq analysis in the buckwheat root tip [20]. The following most abundant categories were ‘transport’, ‘stress/defense response’, and ‘signal transduction’. By contrast, in the downregulated DEGs, genes related to ‘protein translation, processing and degradation’ constituted the most affected category, which is in accordance with our previous study in rice bean [23]. Following were genes involved in ‘metabolism’, ‘transcription regulation’ and ‘stress/defense response’. Except for the downregulated genes involved in ‘protein translation, processing and degradation’, genes related to ‘metabolic process’ were the most affected by Al stress both in up- and downregulated genes, suggesting that buckwheat leaves rapidly reprogram their metabolism to cope with Al toxicity.

### 2.4. Genes Involved in Protein Translation, Processing and Degradation

The proportion of genes associated with ‘protein translation, processing and degradation’ dramatically differentiates the upregulated and downregulated genes (Figure 1). Particularly prominent are genes encoding ribosomal proteins, which are almost exclusively downregulated by Al stress. For example, the expression of 284 genes that encode ribosomal proteins was reduced, whereas only four related genes displayed the opposite pattern (Table S2). On the other hand, genes associated with protein degradation, including protein ubiquitination and proteolysis, were relatively more abundant in upregulated genes than downregulated genes. In accordance with the present results, our previous transcriptional analysis of the rice bean root apex also demonstrated that genes related to ‘protein translation, processing and degradation’ were substantially downregulated by moderate Al stress, in which root elongation was not significantly affected [23]. It appears that the reprogramming of protein metabolism represents a flexibility of plants in response to Al stress.

### 2.5. Genes Involved in Transcription Regulation

Transcriptional responses of plants to environmental stresses have been extensively investigated, and TFs regulate the expression of genes at the transcriptional level [24]. We identified a total of 49 TF genes (40 upregulated and 9 downregulated) from buckwheat leaf DEGs affected by Al stress (Table 2). The Al-upregulated TFs were categorized into 16 TF families according to the TF family classification proposed by Mitsuda and Ohme-Takagi [24]. By contrast, the downregulated TF genes belong to five TF families. Among these TF genes, a gene (comp30095_c0_seq1) encoding ATAF1 (a NAM, ATAF, and CUC (NAC) TF) was consistently upregulated in both root tips and leaves. However, a gene (comp7947_c0_seq1) encoding a C2H2-like zinc finger TF was downregulated by Al stress in buckwheat leaves. The difference in the transcription regulation of TF genes suggests that transcriptional regulation mechanisms differ between the root apex and leaves under Al stress.

Accumulating evidence suggests that chromatin modification is involved in transcriptional responses [25]. Among 97 downregulated genes involved in transcriptional regulation, 83 were involved in chromatin remodeling and modification. By contrast, only three genes were identified to be involved in chromatin modification out of 50 upregulated transcription regulation genes (Table S2). In our previous RNA-seq analysis of the buckwheat root tip under Al stress, no chromatin remodeling and modification genes were identified [20], suggesting that the chromatin context of transcriptional regulation is specifically involved in the response of buckwheat leaves to Al stress.

### 2.6. Genes Encoding Transporters

**Figure 2:**
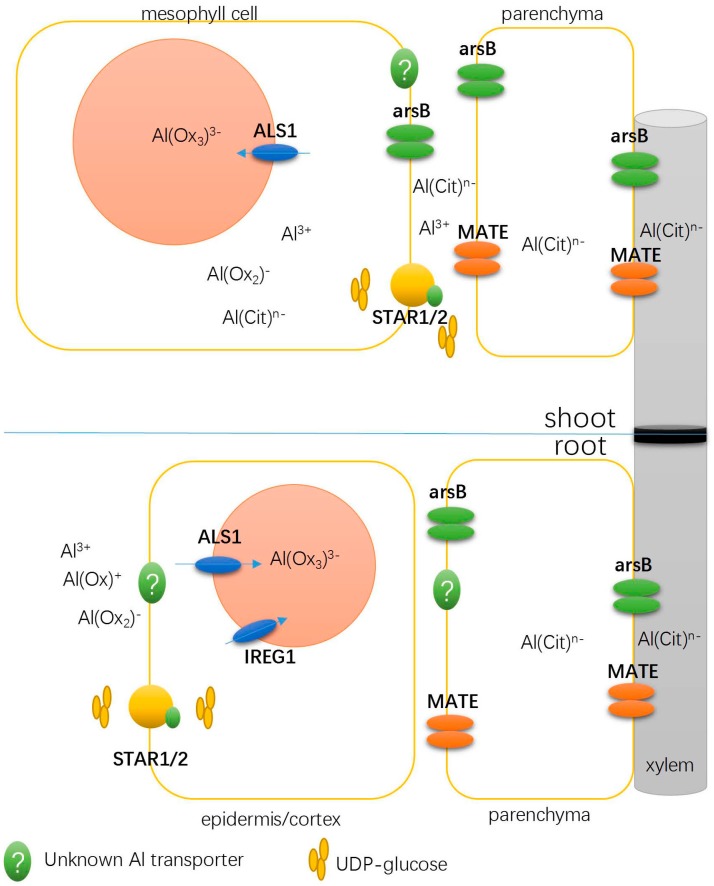
Illustration of Al-upregulated transporter genes that are conserved between roots and leaves. arsB may function as a bidirectional Al transporter. ALS, aluminum sensitive; arsB, a secondary carrier protein of bacterial arsenic resistance (ars) operons; MATE, multidrug and toxic compound extrusion; STAR, sensitive to Al rhizotoxicity.

We identified 100 genes from buckwheat leaves that encode transporters, of which 78 were upregulated by Al stress and 22 were downregulated (Table 3). These transporter proteins have diverse roles according to their potential substrate specificity. Noticeably, several genes were found to be conservatively upregulated in both the root tips and leaves (Table 3; Figure 2). These genes include one encoding a divalent ion symporter, three encoding ALS1, four encoding homologs to AtMATE, two encoding STAR1 and two genes encoding ALS3 (Figure 2). Interestingly, five aquaporin genes belonging to the plasma membrane intrinsic proteins (PIPs), the tonoplast intrinsic proteins (TIPs) and the small basic intrinsic proteins (SIPs) subfamilies of the aquaporin family were found to be downregulated, whereas one gene encoding PIP1;5 was transcriptionally upregulated by Al stress (Table 3). 

### 2.7. Genes Associated with Development

Different from the root, we specifically identified a group of genes associated with ‘development’ (Figure 1; Table S2). Among 25 upregulated and 17 downregulated development-associated genes, a large proportion was supposed to be involved in flowering. 

### 2.8. Coregulated Genes between Roots and Leaves under Al Stress

Some conserved mechanisms must coexist between roots and shoots, because buckwheat detoxifies Al within both roots and leaves. Thus, we searched for genes in the roots and leaves with the same sequence homologies with *Arabidopsis* genes. A total of 56 genes were identified, of which 48 were upregulated by Al stress and 8 were downregulated (Table S3). Of great interest were five genes showing similarity with the transporter genes *ALS1*, *AtSTAR1*, *ALS3*, *AtMATE* and *divalent ion transporter*. While AtSTAR1/ALS3 could protect the cell wall from Al binding [18], ALS1 is important for the compartmentalization of Al within vacuoles [26,27]. The *Arabidopsis* AtMATE protein is involved in citrate secretion, to the root apoplast and rhizosphere, to chelate Al [28]. Because buckwheat roots secrete oxalate instead of citrate, the upregulation of the *AtMATE* homolog gene is likely to facilitate Al translocation in the xylem [29]. Also intriguing is the identification of genes encoding acyl-activating enzyme 3 proteins that have recently been reported to be involved in Al tolerance by regulating cytosolic oxalate homeostasis [30,31]. 

### 2.9. Validation of Gene Expression by qRT-PCR Analysis

We validated the RNA-seq data by using quantitative reverse transcription (qRT)-PCR analysis to measure the expression of 20 DEGs upregulated by Al, and 15 DEGs downregulated by Al. The high correlation coefficient (*R*^2^ = 0.9324) between RNA-seq data and qRT-PCR results (Figure S2) suggests that the RNA-seq data were reliable.

## 3. Discussion

Buckwheat displays high Al resistance by employing three different strategies: root tip Al exclusion, Al tolerance based on Al sequestration, and Al transport from the root to the shoot for subsequent sequestration in leaf cell vacuoles. While recent RNA-seq studies on buckwheat roots have greatly improved our understanding of root Al exclusion and tolerance mechanisms, little is known about leaf Al sequestration [17,20,32]. In the present study, we identified a total of 33,931 unigenes from buckwheat leaves, thus providing a platform facilitating future gene function characterization (Table 1). In addition, we identified 1063 upregulated and 944 downregulated genes after exposure of buckwheat to 20 µM Al for 24 h. These DEGs were functionally categorized into a variety of physiological and molecular events (Figure 1), which revealed conserved mechanisms between roots and leaves on the one hand, and distinct adaptive mechanisms in leaves on the other hand.

### 3.1. Detoxifying Apoplastic Al with a Bacterial-Type ABC Transporter

We identified two genes homologous to OsSTAR1/AtSTAR1 and two genes homologous to OsSTAR2/ALS3 (Table 3). Consistent with our present study, a previous RNA-seq analysis of buckwheat leaves identified a gene encoding FeSTAR1 that was upregulated by Al stress [17]. In rice, OsSTAR1 and OsSTAR2 form a bacterial-type ATP-binding cassette (ABC) transporter to transport uridine diphosphate (UDP)-glucose for the modification of cell walls, which detoxifies Al in the apoplast [18]. Recently, experimental evidence has indicated that *Arabidopsis* AtSTAR1 and ALS3 could also interact with each other to form a bacterial-type ABC transporter [33]. Given FeSTAR1 and FeALS3 need to work together with respect to Al resistance, it is not surprising in this study that not only FeSTAR1, but also FeALS3, were identified to be coregulated by Al stress. In addition, it is interesting that the expression of *FeSTAR1* and *FeSTAR2* was induced in both the roots and leaves, which is different from the expression pattern found in rice and *Arabidopsis* [18,34]. Unlike rice and *Arabidopsis*, buckwheat accumulates Al within leaves [15] (Figure S1). Thus, it is very likely that the FeSTAR1/FeALS3 protein complex is also involved in protecting the leaf cell apoplast from Al injury.

### 3.2. Transporters Possibly Involved in Xylem Al Unloading and Sequestration

Sequestration of Al within buckwheat leaf vacuoles involves xylem Al unloading and sequestration into vacuoles across the tonoplast, both of which require the participation of transporters. Unlike in roots, where Al was mostly bound to the cell wall [35], Al in leaves resides mainly in the symplasm [36,37]. Such a difference in Al distribution between the roots and leaves is not surprising, because Al is present in the form of an Al-citrate complex after xylem loading [38]. Thus, it appears that Al is actively transported into leaf cells in the form of an Al-citrate complex. Here, we identified an Al-upregulated gene (comp31346_c0_seq4) encoding a putative divalent ion symporter that is predicted to be a member of the anion permease ArsB/NhD family. Recently, Negishi et al. identified an Al transporter gene, *HmPALT2*, from the hydrangea sepal, which displays high sequence similarity with the *Arabidopsis* divalent ion symporter AT1G02260. Functional characterization of HmPALT2 revealed that it can transport Al in the form of Al^3+^ and Al-citrate complexes [39]. High sequence similarity between comp31346_c0_seq4 and HmPALT2 suggests that this gene is a strong candidate for xylem Al unloading. More intriguingly, in a previous RNA-seq analysis of the buckwheat root apex, we identified three upregulated genes encoding the same divalent ion symporters (Table S3; Figure 2), suggesting conserved mechanisms of Al transport between roots and leaves through transcriptional regulation of this gene. The functional characterization of this gene is currently underway.

Once entering leaf cells, Al is sequestrated into vacuoles [37]. Because almost all the Al in the protoplast was found to be present in vacuoles [36], there must be a tonoplast-localized transporter that actively transports Al into vacuoles. We identified three upregulated genes showing high sequence similarity with ALS1 that could fulfil this function (Table S2). Both rice OsALS1 and *Arabidopsis* ALS1 are tonoplast-localized transporters that are involved in the sequestration of Al into the vacuole [26,27]. In the buckwheat root tip, we identified a gene encoding FeALS1 and a gene encoding FeIREG1, which is consistent with a previous report [17,20]. Very recently, based on the proteomic analysis of a tonoplast-rich fraction, Lei et al. identified two FeALS1 proteins—FeALS1.1 and FeALS1.2—from buckwheat leaves, and demonstrated their function as tonoplast-localized transporters for Al sequestration into vacuoles [40]. While the expression of *FeALS1.1* was induced by Al both in the roots and shoots, *FeALS1.2* was constitutively expressed. However, considering the frequent occurrence of gene duplication events in this cross-pollinating species, it is not surprising that we have identified three ALS1 homologs whose expression was induced by Al [20] (Table S2). At present, it remains unknown why FeIREG1 is only involved in root Al sequestration [41], while FeALS1 is conserved between roots and leaves. One plausible explanation may be that FeALS1 is not sufficient for Al sequestration into root vacuoles, and thus an additional transporter, FeIREG1, is required. However, FeALS1 is sufficient for Al sequestration in leaves. Our finding, that there are three homologs of ALS1 involved in buckwheat leaf defense against Al stress, supports our current supposition.

Recently, Negishi et al. identified tonoplast- and plasma membrane-localized Al transporters, HmVALT1 and HmPALT1, from the sepals of the hydrangea, both of which belong to the aquaporin protein family [42]. HmVALT and HmPALT1 belong to the tonoplast-intrinsic proteins (TIPs) and nodulin 26-like intrinsic proteins (NIPs) of aquaporin, respectively. Most recently, NIP1;2 was reported to be a plasma membrane-localized transporter that facilitates Al–malate transport from the root cell wall into the root symplasm [43]. Here, only one gene (comp29405_c0_seq1) displaying sequence similarity with *Arabidopsis* PIP1;5 was found to be upregulated by Al (Table 3). Clearly, FePIP1;5 belongs to a divergent subfamily of the aquaporin family, and is unlikely to be involved in Al transport. In accordance with this work, our previous study showed that no aquaporin genes were upregulated in the buckwheat root tip by Al stress [20].

### 3.3. Flowering Regulation in Response to Al Stress

We found that a subset of genes potentially related to flowering are differentially regulated by Al stress. In *Arabidopsis*, different pathways converge on a few floral integrator genes such as *flowering locus T* (*FT*) that promote flowering. During long days, the expression of *FT* is upregulated by the TF constans (CO), and the FT protein is moved from mature leaves to the meristem to initiate flowering. In the present study, a total of eight upregulated and three downregulated TF genes were found to be associated with flowering (Table 2). For example, one *constans-like 4* and two *constans-like 5* genes were upregulated by Al stress. Furthermore, a bHLH TF gene encoding HCEATE that is involved in floral organ development was also upregulated by Al stress [44]. However, a gene encoding cycling DOF factor 3 (CDF3), that functions as a transcriptional repressor of the floral integrator genes CO and FT, was also upregulated [45]. In addition to delaying flowering, it was recently reported that *Arabidopsis* CDF3 is also involved in the mechanisms of tolerance to drought and low temperature [46]. Similarly, the expression of a *golden2-like 1* gene that negatively regulates flower development was also inhibited by Al stress. It appears that Al stress differentially affects the expression of flowering-related TFs in buckwheat leaves.

In the upregulated genes, we identified 3 *UDP-glucosyl transferase 87A2 (UDPG87A2)* genes, five *similar to flowering promoting factor1* genes, two *ELF4-like* genes and two *flowering promoting factor1 (FPF1)* genes. By contrast, two *jasmonate-zim-domain protein1* genes, one *calmodulin-like 24* gene, three *heat shock protein 90.2* genes, two *ELF4* genes, four *nucleostemin-like 1* genes and four *frigida-like protein* genes were identified in the downregulated genes. Among them, *UGT87A2*, *FPF1*, *similar to flowering promoting factor 1* and *nucleostemin-like 1* function as positive regulators that promote flowering [43,47], while the *ELF4-like 4* and *frigida-like protein* genes serve as repressors of flowering, and others may indirectly affect flowering [48,49]. These results suggest that flowering time, or the transition from vegetative to reproduction growth, is affected by Al stress. Clearly, more work is required to understand how buckwheat integrates the Al stress response and flowering time. It is becoming increasingly evident that altering flowering time is an evolutionary strategy by which plants maximize the chances of reproduction under stress conditions. For example, nitrate deficiency promotes flowering in *Arabidopsis* [50,51]. Under drought stress, plants often accelerate the flowering process to speed life history, thereby avoiding drought [52,53]. Therefore, in the future, it will be interesting to know whether or not buckwheat adapts to Al toxicity by regulating flowering time.

### 3.4. Chromatin Regulation in Response to Al Stress

We conclude that chromatin regulation may play an important role in the response of buckwheat leaves to Al stress. In addition to the TFs that play a pivotal role controlling gene expression, a large proportion of genes were involved in chromatin regulation, especially among the downregulated DEGs (Table S2). For example, there are 18 genes encoding histone 3.1 and 18 genes encoding histone superfamily protein, respectively; 11 genes encoding histone H2A 12; and 4 genes encoding histone H2A 10 and H2B, respectively. Each nucleosome is an octamer composed of two copies of histones, H2A, H2B, H3 and H4, that package eukaryotic DNA into repeating nucleosomal units. Accumulating evidence suggests that changes in chromatin structure are involved in the regulation of gene expression in response to abiotic stress [25]. Thus, the conspicuous downregulation of a large group of genes related to chromatin remodeling must have a specific role in the response of buckwheat leaves to Al stress, although this requires further investigation.

We also identified genes associated with the post-translational modification of histone N-tails. Al stress depressed the expression of three *histone deacetylase 2C* genes and two *histone deacetylase 3* genes (Table S2). Histone acetylation reduces charge interaction between histones and DNA, thereby facilitating transcription activation. Kim et al. found that some drought-responsive genes such as *RD29A*, *RD29B*, *RD20* and *RAP2.4* were differentially acetylated at K9, K14, K23 and K27 of histone 3 in *Arabidopsis* under short-term drought stress, and the histone acetylation level was positively correlated with gene expression [53]. The level of H3K9Ac in *RD20* and *RD29A* was quickly reduced after rehydration, which was correlated with gene expression reduction [54]. Apparently, the downregulation of histone deacetylase genes may play an important role in regulating gene expression under Al stress. However, the specific target genes of histone acetylation, and the corresponding phenotypic consequences, have yet to be investigated.

In contrast, a gene encoding histone H3K4-specific methyltransferase was found to be upregulated by Al stress (Table S2). A potential link between histone methylation and gene expression under abiotic stress is emerging. For example, Zong et al. reported that the H3K4me3 modification level was significantly and positively correlated with the transcript level in rice under drought stress, although only 13% of stress-responsive genes were differentially affected by H3K4me3 methylation [55]. A genome-wide study of H3K4 methylation in *Arabidopsis* plants exposed to drought stress also revealed that H3K4me3 methylation is positively correlated with transcript level [56,57]. Consistent with the downregulation of histone deacetylase genes, the upregulation of histone H3K4-specific methyltransferase is associated with the upregulation of stress-responsive genes, which may be critical for the adaptation of buckwheat leaves to Al stress.

In summary, we provided the transcriptome information for buckwheat leaves in response to Al stress. Functional classification of both up- and downregulated genes, according to the biological processes of homologous genes in *Arabidopsis*, revealed distinct mechanisms by which buckwheat leaves cope with Al stress. Moreover, comparative analysis of transporter genes between roots and shoots not only revealed the conserved molecular basis of Al resistance between roots and leaves, but also provided us with a basis for the characterization of novel Al resistance genes. 

## 4. Materials and Methods

### 4.1. Plant Materials and Growth Conditions

Seeds of an Al-tolerant buckwheat (*Fagopyrum esculentum* Moench cv. Jiangxi, Jiangxi province, China) were germinated and cultured according to our previous work [20]. On day 3, uniform seedlings were transferred to 1/5 strength Hoagland nutrient solution (pH 5.5) consisting of KNO_3_ (1.0 mM), Ca(NO_3_)_2_ (1.0 mM), MgSO_4_ (0.4 mM) and (NH_4_)H_2_PO_4_ (0.2 mM), and the micronutrients NaFeEDTA (20 µM), H_3_BO_3_ (3.0 µM), MnCl_2_ (0.5 µM), CuSO_4_ (0.2 µM), ZnSO_4_ (0.4 µM) and (NH_4_)_6_Mo_7_O_24_ (1 µM). The solution was renewed every 3 d. When the true leaf was fully expanded, the seedlings were exposed to the same nutrient solution with (NH_4_)H_2_PO_4_ concentration decreased to 10 µM either in the absence (−Al) or presence (+Al) of 20 µM Al for 24 h. After treatment, the expanded true leaf was collected and immediately frozen in liquid nitrogen for future use. Plants were cultured in an environmentally controlled growth room with a 14 h/26 °C day (light intensity of 300 µmol photons m^−2^·s^−1^) and a 10 h/22 °C night regime.

### 4.2. Determination of Al Accumulation

In order to determine Al accumulation in buckwheat, an Al-resistant rice bean, which is a known Al excluder, was used as a reference [19]. Seeds of rice bean and buckwheat were germinated and cultured following the same procedure. Two-week-old seedlings were exposed to the 1/5 strength nutrient solution with 10 µM (NH_4_)H_2_PO_4_ either in the absence (−Al) or presence (+Al) of 20 µM Al for 24 h. After treatment, roots and shoots were collected and dried at 70 °C in an oven for 2 days. The Al concentration was measured by inductively coupled plasma mass spectrometry after digestion of samples with HNO_3_.

### 4.3. RNA Isolation and Solexa Sequencing

The total RNA isolation and sequencing procedures followed our previous work [20]. 

### 4.4. Sequence Assembly and Annotation

Sequence assembly and annotation were the same as our previous work [20]. The sequences of all unigenes were deposited in the NCBI database (accession number: SRA589522).

### 4.5. Differential Gene Expression Analysis and Gene Ontology Biological Processes Analysis

The reads per kb million reads (PRKM) method was used to calculate gene expression levels [22]. To identify Al-induced differentially expressed genes, log_2_ (fold change) ≥ 1 and RPKM values ≥10 were set as the cut-off. For each group of DEGs, gene ontology (GO) biological processes analysis was performed according to our previous study [20].

### 4.6. Quantitative RT-PCR Analysis

The total RNA extraction qRT-PCR procedures followed our previous work [20]. Primers for qRT-PCR analysis are listed in Table S4.

## Figures and Tables

**Table 1:** Summary for the buckwheat leaf transcriptome in control (−Al) and Al-treated (+Al) libraries.

Assembly Summary	−Al	+Al
Total number of reads	89,531,252	59,073,878
Total base pairs (bp)	7,162,500,160	4,725,910,240
Average read length (bp)	80	80
Total number of transcripts	50,782	
Mean length of transcripts	716	
Total number of unigenes	33,931	
Mean length of unigenes	677	
Sequence with *E*-value	26,300	

**Table 2:** Al-regulated genes encoding transcription factors.

Gene ID	Fold Change	TAIR ID	Description	Category ^1^	Biological Processes ^2^
Upregulated					
comp24276_c0_seq2	1.391	AT1G46768	Related to AP2 1	AP2-EREBP	Ethylene-activated signaling pathway, response to cold, response to water deprivation
comp28160_c0_seq1	1.029	AT4G36900	Related to AP2 10	AP2-EREBP	Ethylene-activated signaling pathway
comp27009_c0_seq1	1.045				
comp4672_c0_seq1	1.329	AT1G76110	HMG (high mobility group) box protein with ARID/BRIGHT DNA-binding domain	ARID	Karyogamy, polar nucleus fusion
comp13872_c0_seq1	1.257				
comp28434_c0_seq1	1.029	AT5G49700	AT-HOOK motif nuclear localized protein 17	AT-hook	Unknown
comp20160_c0_seq1	1.651	AT5G43700	IAA4	AUX/IAA	Response to auxin
comp32831_c0_seq1	1.326				
comp27076_c0_seq2	1.084	AT1G09530	Phytochrome interacting factor 3	bHLH	De-etiolation, gibberellic acid-mediated signaling pathway, positive regulation of anthocyanin metabolic process, red or far-red light signaling pathway
comp17528_c0_seq2	1.750	AT3G19860	BHLH121	bHLH	Unknown
comp2972_c0_seq1	1.898				
comp28503_c0_seq1	1.511	AT5G61270	Basic helix-loop-helix (bHLH) phytochrome interacting factor	bHLH	De-etiolation, red, far-red light phototransduction
comp16875_c0_seq1	1.294	AT5G67060	Encodes a bHLH transcription factor	bHLH	Carpel formation, gynoecium development, regulation of auxin polar transport, transmitting tissue development
comp24549_c0_seq1	1.005	AT3G17609	HY5-homolog	bZIP	Red, far-red light phototransduction, response to UV-B, response to karrikin
comp21671_c0_seq1	1.015				
comp31175_c0_seq1	1.074	AT5G28770	Basic leucine zipper 63	bZIP	Response to abscisic acid, response to glucose
comp30394_c0_seq1	1.113				
comp18077_c0_seq1	1.106	AT1G68520	B-Box domain protein 14	C2C2-CO-LIKE	Unknown
comp31981_c0_seq1	1.432	AT5G24930	Constans-like 4	C2C2-CO-LIKE	Regulation of flower development
comp31991_c0_seq2	1.037	AT5G57660	Constans-like 5	C2C2-CO-LIKE	Regulation of flower development
comp31991_c0_seq1	1.291				
comp5736_c0_seq1	1.243	AT3G21270	DOF zinc finger protein 2	C2C2-Dof	Unknown
comp13725_c0_seq1	1.216				
comp60617_c0_seq1	1.144	AT3G47500	Cycling DOF factor 3	C2C2-Dof	Flowering
comp27353_c0_seq1	1.010	AT2G05160	CCCH-type zinc finger family protein with RNA-binding domain	C3H	Unknown
comp30562_c0_seq1	1.351	AT2G20570	Golden 2-like 1	G2-like	Chloroplast organization, negative regulation of flower development, negative regulation of leaf senescence, regulation of chlorophyll biosynthetic process
comp32323_c0_seq1	1.064	AT3G04450	Homeodomain-like superfamily protein	G2-like	Unknown
comp25041_c0_seq1	1.177	AT2G46680	Homeobox 7	HB	Response to abscisic acid, response to water deprivation
comp28163_c0_seq1	1.160				
comp32393_c0_seq2	1.239	AT5G04760	Duplicated homeodomain-like superfamily protein	MYB	Unknown
comp110794_c0_seq1	1.074	AT2G46830	Circadian clock-associated 1	MYB-related (flower)	Flowering
comp80633_c0_seq1	1.173				
comp30095_c0_seq1	1.245	AT1G01720	ATAF1	NAC	Negative regulation of abscisic acid-activated signaling pathway
comp47425_c0_seq1	2.096	AT3G15500	ANAC055	NAC	Jasmonic acid-mediated signaling pathway, response to water deprivation
comp26124_c0_seq1	2.605	AT3G15510	ANAC056	NAC	Jasmonic acid-mediated signaling pathway, response to water deprivation
comp27368_c0_seq1	1.295	AT1G32700	PLATZ transcription factor family protein	PLATZ	Unknown
comp28592_c0_seq2	1.021	AT4G17900	PLATZ transcription factor family protein	PLATZ	Unknown
comp83456_c0_seq1	1.048	AT4G02020	SET domain-containing protein 10	SET	Endosperm development
comp94475_c0_seq1	1.981	AT5G13080	WRKY75	WRKY	Atrichoblast differentiation, lateral root development, induced by Pi starvation
comp87094_c0_seq1	2.190				
Downregulated					
comp23084_c0_seq1	−1.004	AT4G34590	Basic leucine-zipper 11	bZIP	Response to sucrose
comp7947_c0_seq1	−1.088	AT1G75710	C2H2-like zinc finger protein	C2H2	Unknown
comp18294_c0_seq1	−1.002	AT5G28640	GRF1-interacting factor 1	GIF	Adaxial/abaxial pattern specification, leaf development
comp30187_c0_seq2	−1.260	AT4G24540	Agamous-like 24	MADS	Flowering
comp30099_c0_seq1	−1.224				
comp30187_c0_seq1	−1.219				
comp17187_c0_seq1	−1.042	AT1G73230	Nascent polypeptide-associated complex NAC	TF_GTF	Response to salt stress
comp27260_c0_seq1	−1.051				
comp27190_c0_seq1	−1.004				

^1^ Transcription factor category was assigned based on Mitsuda and Ohme-Takagi [24]; ^2^ biological processes were assumed according to TAIR gene ontology (GO) analysis.

**Table 3:** Al-regulated genes that encode putative transporters.

Gene ID	Fold Change	TAIR ID	Description	Transport Substrate ^1^
Upregulated				
comp31346_c0_seq4	1.044	AT1G02260	Divalent ion symporter	Al
comp29520_c0_seq1	1.250	AT5G39040	Aluminum-sensitive 1	Al
comp3719_c0_seq1	1.065			
comp32696_c0_seq2	1.060			
comp18915_c0_seq1	1.333	AT2G41190	Transmembrane amino acid transporter family protein	Amino acid
comp14037_c0_seq1	1.054			
comp18529_c0_seq1	1.266	AT4G01100	Adenine nucleotide transporter 1	AMP; ADP; ATP
comp3758_c0_seq1	1.016	AT5G17400	Endoplasmic reticulum-adenine nucleotide transporter 1	AMP; ADP; ATP
comp29175_c0_seq1	1.263	AT5G15410	Cyclic nucleotide gated channel 2	Ca^2+^
comp29835_c0_seq1	1.124			
comp15571_c0_seq1	1.857	AT2G23790	Protein of unknown function (DUF607)	Ca^2+^
comp9425_c0_seq1	1.860			
comp68193_c0_seq1	1.851			
comp31329_c0_seq1	1.403	AT5G61520	Major facilitator superfamily protein	Carbohydrate
comp27968_c0_seq1	1.499			
comp29178_c0_seq1	1.403	AT5G18840	Major facilitator superfamily protein	Carbohydrate
comp7291_c0_seq1	1.167			
comp23114_c0_seq1	1.510			
comp98352_c0_seq1	1.678	AT1G51340	MATE efflux family protein	Citrate
comp11267_c0_seq1	2.039			
comp96590_c0_seq1	1.764			
comp88250_c0_seq1	1.705			
comp81877_c0_seq1	1.967	AT3G21090	ABCG15	Cutin
comp56152_c0_seq1	2.033			
comp96281_c0_seq1	2.336			
comp22596_c0_seq2	1.350	AT5G16150	Putative plastidic glucose transporter	Glucose
comp22596_c0_seq1	1.314			
comp25276_c0_seq1	1.121			
comp49196_c0_seq1	2.598	AT3G47960	NRT1/ PTR family 2.10	Glucosinolate
comp26699_c0_seq1	2.651			
comp28002_c0_seq1	2.654			
comp91186_c0_seq1	3.385			
comp24147_c0_seq1	1.022	AT4G24120	Yellow stripe-like 1	Iron-nicotianamine; oligopeptide
comp106142_c0_seq1	1.425	AT1G31120	K^+^ uptake permease 10	K^+^
comp30691_c0_seq1	1.012	AT2G35060	K^+^ uptake permease 11	K^+^
comp12992_c0_seq1	2.012	AT3G02850	Stellar K^+^ outward rectifier	K^+^
comp16659_c0_seq1	1.954	AT5G37500	Gated outwardly-rectifying K^+^ channel	K^+^
comp84025_c0_seq1	2.025			K^+^
comp3908_c0_seq1	1.328	AT5G46240	Potassium channel protein (KAT1)	K^+^
comp69280_c0_seq1	1.332	AT3G18830	Polyol/monosaccharide transporter 5	Linear polyols; myo-inositol; monosaccharides
comp7769_c0_seq1	1.787	AT4G21120	Cationic amino acid transporter 1	Lys, Arg and Glu
comp10794_c0_seq1	1.790			
comp13115_c0_seq1	1.429			
comp8282_c0_seq1	1.398			
comp25372_c0_seq1	1.911			
comp32005_c0_seq2	1.502	AT1G25480	Aluminum-activated malate transporter family protein	Malate
comp33002_c0_seq1	1.168	AT1G77210	Sugar transport protein 14	Monosaccharide
comp5166_c0_seq1	1.145	AT3G54140	Peptide transporter 1	Peptide
comp15597_c0_seq1	1.121	AT2G38940	Phosphate transporter 1;4	Pi
comp14809_c0_seq1	1.128			
comp32347_c0_seq1	1.111	AT3G26570	Phosphate transporter 2;1	Pi
comp22508_c0_seq1	1.293	AT5G43350	Phosphate transporter 1	Pi
comp26614_c0_seq1	1.078	AT5G54800	Glucose 6-phosphate/phosphate translocator 1	Pi; PEP, triose phosphate; glucose 6-phosphate
comp30882_c0_seq12	1.154	AT4G18210	Purine permease 10	Purine; purine derivatives
comp30860_c0_seq2	1.241			
comp48026_c0_seq1	1.237	AT1G71880	Sucrose-proton symporter 1	Sucrose
comp18524_c0_seq1	1.234	AT5G19600	Sulfate transporter 3;5	Sulfate
comp25104_c0_seq5	1.224	AT3G23560	Aberrant lateral root formation 5	Toxins
comp25104_c0_seq4	1.197			
comp25244_c0_seq5	1.140			
comp25244_c0_seq2	1.123			
comp26932_c0_seq1	2.705	AT4G30420	Usually multiple acids move in and out transporter 34	Unknown
comp69634_c0_seq1	1.345	AT4G36670	Polyol/monosaccharide transporter 6	Glucose; hexose
comp29397_c0_seq1	1.032	AT5G64410	Oligopeptide transporter 4	Oligopeptide
comp25749_c0_seq1	3.018	AT1G67940	AtSTAR1	UDP-glucose
comp26709_c0_seq1	2.987			
comp30641_c0_seq1	2.916	AT2G37330	Aluminum sensitive 3	UDP-glucose
comp30389_c0_seq1	2.852			
comp8633_c0_seq1	1.022	AT5G15640	Mitochondrial substrate carrier family protein	Unknown
comp81941_c0_seq1	1.012			
comp21728_c0_seq1	1.064	AT1G74780	Nodulin-like/major facilitator superfamily protein	Unknown
comp17931_c0_seq1	1.231	AT2G39210	Major facilitator superfamily protein	Unknown
comp5953_c0_seq1	1.419			
comp12616_c0_seq1	1.249			
comp29405_c0_seq1	1.204	AT4G23400	PIP1;5	Water
comp50926_c0_seq1	1.895	AT1G51500	ABCG12	Wax
comp6165_c0_seq1	1.950			
comp92864_c0_seq1	1.903			
Downregulated				
comp29871_c0_seq3	−1.038	AT4G28390	ADP/ATP carrier 3	ADP; ATP
comp30078_c0_seq2	−1.144			
comp30078_c0_seq1	−1.137			
comp29871_c0_seq1	−1.003			
comp31976_c0_seq1	−1.058	AT2G36910	ABCB1/P-glycoprotein 1	Auxin
comp22423_c0_seq1	−1.066	AT1G75500	Usually multiple acids move in and out transporter 5	Auxin
comp28292_c0_seq1	−1.120	AT1G53210	Na^+^/Ca^2+^ exchanger	Ca^2+^
comp33125_c0_seq2	−2.085	AT3G51860	Cation exchanger 3	Ca^2+^ and H^+^
comp23194_c0_seq1	−1.036	AT4G32390	Nucleotide–sugar transporter family protein	Carbohydrate
comp25558_c0_seq1	−1.269	AT5G19760	Encodes a novel mitochondrial carrier	Dicarboxylates; tricarboxylates
comp27413_c0_seq1	−1.285	AT4G27720	Major facilitator superfamily protein	Mo^6+^
comp23196_c0_seq1	−1.057	AT1G64650	Major facilitator superfamily protein	Mo^6+^
comp27697_c0_seq1	−1.264	AT2G26690	NRT1/PTR family 6.2	Oligopeptide
comp32440_c0_seq1	−1.426	AT5G33320	Phosphate/phosphoenolpyruvate translocator	PEP
comp20485_c0_seq1	−1.269	AT5G14040	Mitochondrial phosphate transporter 3	Pi
comp25757_c0_seq1	−1.264	AT3G01550	Phosphoenolpyruvate/phosphate translocator 2	Pi and PEP
comp17525_c0_seq1	−1.012	AT5G62890	Xanthine/uracil permease family protein	Unknown
comp23073_c0_seq1	−2.726	AT4G35100	PIP2;7	Water
comp27116_c0_seq1	−1.065	AT3G16240	TIP2;1	Water
comp24264_c0_seq1	−1.383	AT2G36830	TIP1;1	Water
comp28684_c0_seq1	−1.186	AT3G04090	SIP 1A	Water
comp29502_c0_seq1	−1.146			

^1^ Substrate specificity was assumed according to TAIR GO functional analysis.

## Supplementary Materials

Supplementary materials can be found at www.mdpi.com/1422-0067/18/9/1859/s1.
