# Supplementary material for: Genome-Wide Transcriptome Analysis Reveals Conserved and Distinct Molecular Mechanisms of Al Resistance in Buckwheat (Fagopyrum esculentum Moench) Leaves
Source: Int J Mol Sci. 2017 Aug 27;18(9):1859. doi: 10.3390/ijms18091859 (PMC5618508; doi:10.3390/ijms18091859)
Supplement: Supplementary file 1 [file ijms-18-01859-s001.zip › ijms-218121-supple/ijms-218121-Supplemental Figures-edited-new.pdf]

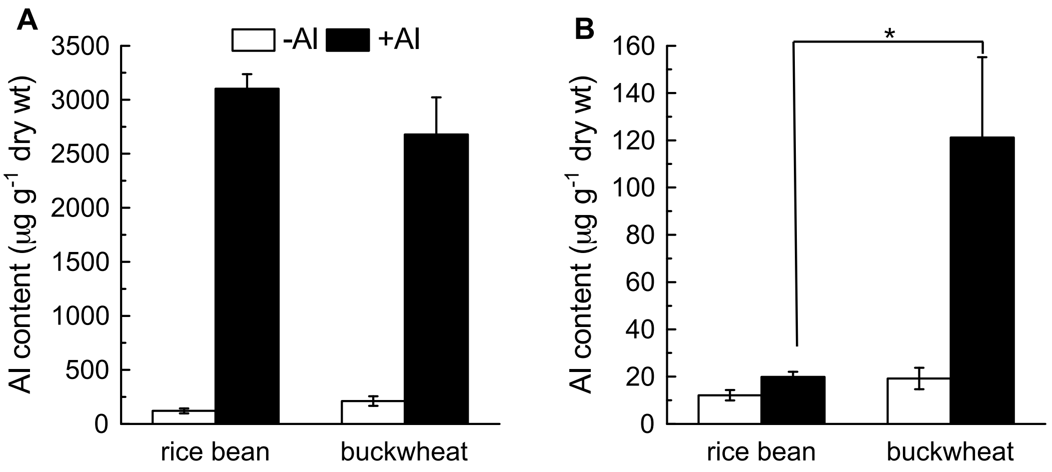

Supplemental Figure S1. Al accumulation in roots and shoots of rice bean and buckwheat. Two-week-old seedlings were exposed to 1/5 strength low-phosphate (10 μM) Hoagland nutrient solution containing 0 or 20 μM Al for 24 h. Al concentration in roots (**A**) and shoot (**B**) were determined by inductively coupled plasma atomic emission spectrometry. Data are means ± SD (n = 4). Asterisk represents statistically significant difference (t-test, $p < 0.05$).

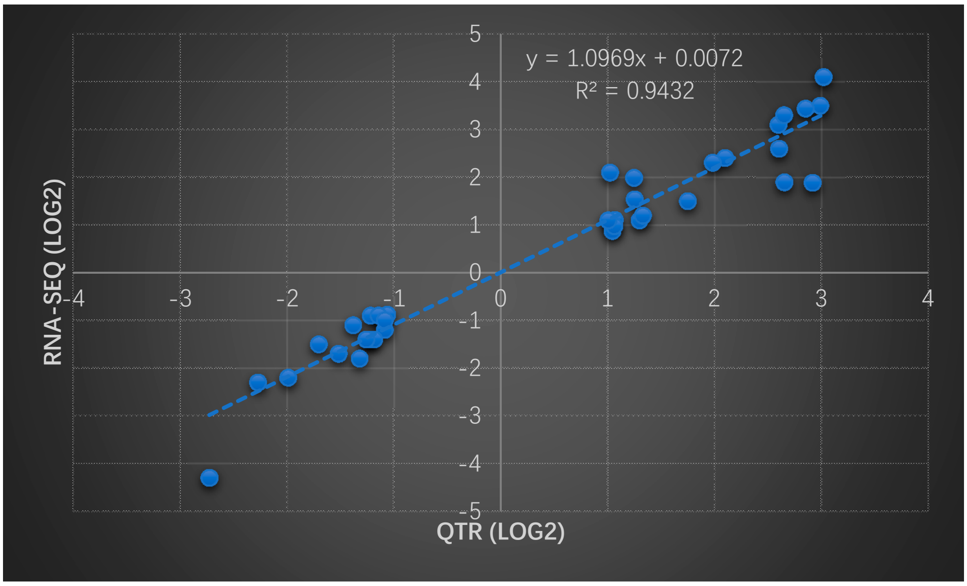

Supplemental Figure S2. Correlation of gene expression levels between RNA-seq data and qRT-PCR analysis. Twenty upregulated and fifteen downregulated genes were selected and subjected to qRT -PCR analysis using the same RNA as for RNA-seq. 18S rRNA was used as an internal standard. RNA-seq data were plotted against data from qRT-PCR. Both x- and y-axes are shown in $\log_2$ scale.
